# Supplementary material for: Characteristics associated with women undergoing their first mammography screening at a younger age
Source: PLOS Glob Public Health. 2025 Nov 13;5(11):e0005458. doi: 10.1371/journal.pgph.0005458 (PMC12614538; doi:10.1371/journal.pgph.0005458)
Supplement: Attachment — Submitted filename: revision.docx [file pgph.0005458.s001.docx]

Thank you for the opportunity to review this important and well-written manuscript. The study addresses a critical gap in the literature by examining the characteristics associated with age at first mammography screening among Kuwaiti women using data from a national screening program. The focus on younger women (40–49 years) is timely given recent updates to screening guidelines and low participation rates in the region. The manuscript is generally well-structured and clearly presented, with thoughtful interpretations.

**That said, I have several comments that, if addressed, could strengthen the clarity, rigor, and interpretability of the findings.**

**Introduction – General comments**

* Make sure references [1]–[5] are up to date, peer-reviewed, and appropriate.

* Line 64–65: Consider rephrasing for readability: “Breast cancer is the most commonly diagnosed cancer and a leading cause of cancer-related death among women worldwide..."

* Line 65: Add a hyphen to "cancer-related".

* Line 66: "Transitioned countries" may be unclear. Please rephrase it.

* Add 1–2 sentences on why incidence is higher in those countries (e.g., reproductive risk factors, screening uptake).

* Add 1–2 sentences on why incidence is higher in those countries (e.g., reproductive risk factors, screening uptake).

*Line 84: author cited [5] (USPSTF) again, but it is not clear how it is directly tied to interventions in Kuwait. Consider removing or replacing with a more regional source, if available.

* Line 92–93: Instead of “versus (vs.) older age (50+ years)”, consider writing smoothly: "...screening at age 40–49 compared to age 50 and older."

**Specific comments**

* Is mammography only available through KNMSP, or are there alternative access points (e.g., private sector, tertiary hospitals)? How might this affect the interpretation of low participation rates in the national program?

**Materials and Methods:**

* The criteria include exclusion for missing screening history data (line 104), but the extent of missingness is not described. How many participants were excluded due to missing screening history responses? Were sensitivity analyses considered to address potential bias?

* How was “first mammography” operationally defined, and was this based solely on self-report or linked medical records?

* Were there any efforts to validate self-reported screening history? How representative are the 5,242 included women of the larger eligible population (e.g., all Kuwaiti women aged 40+)?

* Were participants from all regions of Kuwait equally represented in the KNMSP data used?

* What was the rationale for excluding non-Kuwaiti women?

* Given that a substantial portion of the population in Kuwait consists of non-nationals, could their exclusion limit the generalizability of the findings to the broader population of women living in Kuwait?

* The manuscript would benefit from the inclusion of a participant flow diagram (e.g., Figure 1) outlining the inclusion and exclusion criteria used to arrive at the final analytic sample.

* Post hoc power analyses are not informative when results are already known, and reporting “100% power” is misleading. Instead, authors should focus on confidence intervals and effect sizes in the results section. This section could be omitted.

* It’s unclear what specific outcome or comparison the authors assumed an effect size of 0.5 for (e.g., education levels between age groups?).

* A Cohen’s h of 0.5 represents a large effect, which may not be realistic or relevant to public health applications.

* There is no explanation for choosing 0.5. Was it based on prior studies or clinical significance?

* How were manual corrections to height and weight verified? Were they based on logical rules (e.g., swapped values if height < weight) or cross-validated with other records?

* Were nurses and radiology technicians trained in standardized data entry practices, and was data entry audited for accuracy?

**Results:**

* Table 1: Can the authors provide the raw number of women in each age group (40–49 vs. 50+) for each variable in Table 1? (Currently, only row percentages are reported, which limits interpretation.)

* Figure 1 reports behavioral data (exercise, fatty food, alcohol, smoking), but only exercise shows a significant association and is not included in the multivariate model. Clarify whether these were excluded due to non-significance or high missingness.

* In the "Year of screening" row, was a formal trend test conducted across the five years, or only pairwise differences?

* Table 2: Were all 5,242 women included in the analysis for each variable in Table 2, or do denominators vary due to missing data?

* For variables like "age at menopause" and "breastfeeding duration," how were the categories chosen? Were they based on clinical cutoffs or data distribution?

* Given the age overlap (some women in the 40–49 group may be premenopausal), how was menopausal status determined and validated?

* Given the positive association of parity with early screening and the inverse association of longer breastfeeding, can the authors clarify whether this is due to confounding by age or another unmeasured factor?

* Were any variables excluded from the model due to multicollinearity? If so, which ones? What were the variance inflation factor (VIF) values for retained predictors?

* Did the authors explore interaction terms (e.g., education × awareness, parity × age)? If not, could this be a recommendation for future analysis?

* Given the large confidence interval for social media as a source of awareness (OR = 2.576, 95% CI: 1.28–5.19), is this variable based on a small subgroup? Could the authors report the n for this group to clarify its precision?

**Discussion**

* Is the reported 7.8% uptake from Alkhawari et al., 2021 based on the entire eligible population or only women aged 40–69 enrolled in KNMSP?

* Beyond identifying predictors of early screening, did the study explore reasons for delayed or absent screening in the older group (50+)? If not, would the authors consider this a next step in research or intervention design?

* The finding that social media was associated with early screening is intriguing. Could the authors provide more detail on the type of social media platforms used, or whether these campaigns were part of KNMSP outreach?

* Is there a possibility of reverse causation or selection bias influencing this association (e.g., * older women being referred due to symptoms)?

* Was HRT use more common in younger women due to menopausal timing or healthcare access? Could the authors discuss whether HRT use might serve as a proxy for increased healthcare contact?

* Can the authors clarify how the 8% figure was calculated? Does it refer to 5,242 women out of all Kuwaiti women aged 40–69 during 2014–2019?

* While non-Kuwaiti women are excluded, could the authors briefly describe the size and demographic relevance of this population to Kuwait’s overall breast cancer burden? Might this exclusion limit the generalizability of findings to the broader population of women living in Kuwait?

* Can the authors temper the causal language slightly (e.g., “contributing to earlier detection”)? Given the cross-sectional nature of the data, causal inferences should be made cautiously.

* Are there plans for future follow-up studies to examine whether early screening in this group translates into earlier stage diagnosis or better outcomes?
