# Supplementary material for: Characteristics associated with women undergoing their first mammography screening at a younger age
Source: PLOS Glob Public Health. 2025 Nov 13;5(11):e0005458. doi: 10.1371/journal.pgph.0005458 (PMC12614538; doi:10.1371/journal.pgph.0005458)
Supplement: Attachment — Submitted filename: Response to Reviewers PLOS.docx [file pgph.0005458.s003.docx]

**Response to PLOS GPH Reviewers**

We thank the reviewers and editors for their thoughtful feedback. Below we respond point-by-point. *Reviewer comments are in italics; our responses follow.* A version with highlighted changes is provided.

**Introduction - General**

*Reviewer Comment: Make sure references [1]–[5] are up to date, peer-reviewed, and appropriate.*
**Response:** We revised the opening of the Introduction and updated references to align with current evidence and guidance:

- **Reference 1:** Sung et al. (CA Cancer J Clin 2021) — retained for global burden.
- **Reference 2:** Updated to Smith et al. (CA Cancer J Clin 2018) to reflect reproductive and lifestyle risk factors as well as screening uptake.
- **Reference 3:** Retained Marmot et al. (Br J Cancer 2013), which remains the authoritative independent review on the benefits and harms of breast cancer screening.
- **Reference 4:** Updated to Siu et al. (Ann Intern Med 2016), the USPSTF breast cancer screening recommendation statement.
- **Reference 5:** Added USPSTF Recommendation Statement (JAMA 2024), which lowered the starting age for routine screening from 50 to 40.

Reviewer Comment: Line 64–65 rephrase; add hyphen to “cancer-related”.
**Response:** Rephrased for clarity and added the hyphen (“cancer-related death”). (Introduction)

Reviewer Comment: “Transitioned countries” may be unclear—rephrase.
**Response:** Replaced with “high-income countries.” (Introduction)

Reviewer Comment: Add 1–2 sentences on why incidence is higher (risk factors, screening uptake).
**Response:** Added sentences noting reproductive/lifestyle factors and greater screening uptake as contributors. (Introduction)

Reviewer Comment: USPSTF (line ~84) not clearly justified; consider removing or using a regional source.
**Response:** We kept USPSTF because of the 2024 update to age 40 but clarified that Kuwait and other Gulf programs already target women from age 40, aligning locally. (Introduction).We also added the Kuwait National Mammography Screening Program (Alkhawari et al., 2021) to provide national context and report that 7.8% of the total Kuwaiti female population aged 40–69 participated in the program’s first five years.

Reviewer Comment: Wording “versus (vs.) older age (50+)” could be smoother.
**Response:** Revised throughout to “compared with age 50 and older” or “vs. 50+ years,” for consistency. (Abstract, Introduction, Methods)

Additionaly: We Clarified the knowledge gap and study objective.

We revised the final paragraph of the Introduction to state explicitly that few studies examine characteristics of women undergoing their first mammogram at a younger age, but these findings may not directly apply to Kuwait’s unique healthcare and cultural context and have never been investigated. We specify the objective: to describe these characteristics and assess predictors of first screening at 40–49 vs. ≥50 years.

**Introduction - Specific**

Reviewer Comment: Is mammography only available through KNMSP, or are there alternative routes? How does this affect interpretation of low participation?
**Response:** Addressed. We clarified the screening landscape in Kuwait: KNMSP is the **sole population-based provider of screening mammography for average-risk Kuwaiti women (40–69 years)** and operates across all six governorates. Mammography is available in the private sector; however, these services primarily serve **non-Kuwaiti** women, as KNMSP currently enrolls **Kuwaiti nationals only**. Accordingly, our participation estimate (7.8%) pertains to the **eligible Kuwaiti target population within KNMSP**. We also note that we **excluded women who self-reported prior mammography outside KNMSP**. Comprehensive utilisation data from the private sector are not available, so private screening by non-Kuwaiti residents lies outside our sampling frame and is acknowledged as a **scope limitation**. (Introduction; Methods—Study Design and Participants; Strengths & Limitations)

***Reviewer Comment:*** *Clarify multicollinearity and interaction testing in regression.*
**Response:** Implemented. In **Methods: Statistical Analysis**, we now state that multicollinearity was assessed using variance inflation factors (VIF), and **all VIF values were <2**. In **Results** (preceding Table 3), we added: “No variables were excluded due to multicollinearity (all VIF <2). Exploratory interaction terms (education × awareness; parity × age) were tested and were not statistically significant, so they were not retained.”

**Materials and Methods**

*Reviewer Comment:* *Exclusion for missing screening history may introduce selection bias; sensitivity analyses?*
**Response:** We now report the exact exclusions and final analytic N. We did **not** add sensitivity analyses due to data limitations; we note potential selection effects in the study limitations. (Methods; Strengths & Limitations)

*Reviewer Comment:* *Define “first mammography”; self-report or linkage?*
**Response:** Clarified that “first mammography” refers to first screen **within KNMSP**; women reporting prior mammography outside KNMSP were excluded. We created a verification variable, retained the most complete record per person, and removed duplicates. (Methods: Study Design; Data procedures)

*Reviewer Comment:* *Given age overlap (some 40–49 may be peri/premenopausal), how was menopausal status determined/validated?*
**Response:** Added that menopausal status was **self-reported cessation of menstruation**; some overlap with younger ages is possible, and this is noted in Results. (Methods; Results)

*Reviewer Comment:* *Positive association of parity with early screening—could this be confounding by age or another factor?*
**Response:** We adjusted for multiple covariates and **tested interactions** (e.g., parity×age); none were significant. Residual confounding cannot be excluded and is acknowledged. (Statistical Analysis; Results)

*Reviewer Comment:* *Any variables excluded due to multicollinearity? Provide VIFs.*
**Response:** No variables were excluded for multicollinearity; **all VIFs <2**. (This was noted in the Statistical Analysis and Results)

*Reviewer Comment:* *Explore interaction terms (education×awareness, parity×age)?*
**Response:** Done. **Exploratory interactions were tested** and were not significant; thus not retained. (Results; Table 3 note)

*Reviewer Comment:* *Large CI for social media; report n for this group.*
**Response:** Added **n=94 (1.8%)** and cautioned about precision in both Results and Discussion. (Results; Discussion; Table 1)

**Results**

*Reviewer Comment:* *Trend across screening years?*
**Response:** Yes. We report **Cuzick z=4.56, p<0.001**, and a per-year logistic **OR=1.10 (95% CI 1.06–1.14)**. (Results)

*(Note: Additional table formatting/denominator clarifications were implemented—see “Additional revisions not explicitly requested” below.)*

**Discussion**

*Reviewer Comment:* *Clarify what the 7.8% uptake (Alkhawari et al., 2021) represents.*
**Response:** Clarified that **7.8%** refers to the **total Kuwaiti female population aged 40–69**, not only women already in KNMSP. (Added in the Introduction and Discussion)

*Reviewer Comment:* *Beyond predictors of early screening, discuss reasons for delayed/absent screening among ≥50.*
**Response:** Added a paragraph outlining potential reasons (comorbidities, perceived benefit, logistics) and the need for qualitative work. (Discussion)

*Reviewer Comment:* *Social media finding—platforms? KNMSP outreach? Selection/reverse causation?*
**Response:** We note the small n and wide CI; we highlight that this likely reflects **KNMSP-linked campaigns** and self-selection by health-motivated women; **reverse causation cannot be excluded**. We however, lack platform-specific data. (Discussion)

*Reviewer Comment:* *Possibility of selection bias (e.g., older symptomatic referrals)?*
**Response:** The analytic cohort is **asymptomatic women undergoing first KNMSP screening**, which reduces (but does not eliminate) this concern; we flag the limitation. (Methods; Discussion)

*Reviewer Comment:* *Was HRT use more common in younger women due to menopausal timing or healthcare access?*
**Response:** We interpret HRT use as a **proxy for increased healthcare contact** and suggest embedding screening prompts in menopause clinics. (Discussion)

*Reviewer Comment:* *Clarify how the “~8%” figure was calculated.*
**Response:** We state that **5,242 women ≈8%** of Kuwaiti women aged 40–69 during 2014–2019 based on census estimates. (Methods; Discussion)

*Reviewer Comment:* *Briefly describe non-Kuwaiti population and generalizability.*
**Response:** Expanded limitations: non-Kuwaiti residents are a large share of Kuwait’s population; KNMSP targets nationals; exclusion limits generalizability. (Strengths & Limitations)

*Reviewer Comment:* *Temper causal language given cross-sectional design.*
**Response:** We softened causal phrasing throughout (e.g., “may support,” “may encourage,” “potential value”) and explicitly note the design limitation. (Discussion; Conclusion)

*Reviewer Comment:* *Plans for follow-up to assess stage/outcomes?*
**Response:** Added forward-looking statements: **longitudinal evaluation** is needed to determine whether earlier first screening translates into down-staging and improved outcomes. (Conclusion)

**Change to Abstract (author-initiated)**

**Rationale:** We revised the abstract to improve precision and alignment with PLOS Global Public Health guidance that abstracts summarize key quantitative findings. The updated version (≤300 words) now reports effect estimates with 95% CIs and p-values, clarifies the cohort and setting, notes the secular trend, and uses non-causal language.

**What changed:**

- Added sample size, population, and program context (asymptomatic Kuwaiti women ≥40, first screen within KNMSP, 2014–2019).
- Reported adjusted odds ratios with 95% CIs and p-values for primary predictors, including the small social media subgroup (n=94, 1.8%).
- Included the per-year trend (Cuzick z and per-year aOR).
- Tightened the conclusion to emphasize implications, not causation.
- Kept the abstract under 300 words.

**Additional revisions not explicitly requested (for transparency)**

- Converted Tables 1 and 2 to show both counts and **row** percentages by age group, and updated text to match.
- Standardized table footnotes to state that denominators vary due to missing data and that percentages use non-missing responses.
- Clarified KNMSP scope and data capture in Introduction, Methods, and Limitations (KNMSP enrolls Kuwaiti nationals; private-sector screening is not centrally recorded).
- Specified eligible denominators for subset variables (for example, HRT duration among HRT users, menopause age among menopausal women, breastfeeding duration among those who breastfed).
- Added a statement in Methods to clarify the basis for category cutoffs: breastfeeding duration groupings were chosen for both clinical relevance and observed distribution, while menopausal age categories followed standard clinical definitions from prior epidemiological literature.
- Documented model diagnostics and specification choices (all VIF <2; Hosmer–Lemeshow p=0.834; exploratory interactions tested and not retained).
- Tempered language throughout Discussion and Conclusion to avoid causal inference and to propose prospective evaluation.
- **Statistical reporting:** Added both the **Cuzick trend test** and **per-year OR** for trend analysis by screening year
- **Editorial consistency:** Minor copyedits (e.g., hyphenation, consistent labels such as “Ovary removal,” standardized p-value notation).

We appreciate the reviewers’ thoughtful feedback. These revisions improve clarity, methodological transparency, and alignment between text and tables.
