# Supplementary material for: Characteristics associated with women undergoing their first mammography screening at a younger age
Source: PLOS Glob Public Health. 2025 Nov 13;5(11):e0005458. doi: 10.1371/journal.pgph.0005458 (PMC12614538; doi:10.1371/journal.pgph.0005458)
Supplement: Attachment — Submitted filename: Response_to_Reviewers_PLOS_auresp_2.docx [file pgph.0005458.s004.docx]

**Response to Editorial Comments – PLOS Global Public Health**

**Manuscript ID:** PGPH-D-25-01572R1
**Title:** *Characteristics associated with women undergoing their first mammography screening at a younger age*

We thank the Academic Editor, Dr. Titilayo A. Olaoye, for the constructive editorial feedback provided in the decision letter dated **11 October 2025**.
Reviewer #1 confirmed that *all previous comments were fully addressed* and stated that the manuscript was acceptable with no further concerns.

Below we provide our detailed responses to the **editorial suggestions** outlined in the most recent decision letter.

| Editorial Comment | Author Response / Action Taken |
| --- | --- |
| Abstract – Condense wording and rephrase the final sentence (“Targeted, culturally sensitive outreach … ”). Reduce numerical detail to highlight key findings. | The abstract was rewritten and shortened to under 300 words. The final line now reads: “Targeted, culturally tailored outreach … may promote earlier participation.” Numerical details were limited to principal results only. |
| Introduction – Clarify rationale for focusing on first-time screening at younger age and link this to prevention/down-staging. | Added a sentence explicitly linking early screening to prevention and down-staging. The study objective now clearly states the focus on first-time screening among women aged 40–49. |
| Line 91–94: Support “7.8 % screened …” with a citation. | Added citation (Alkhawari et al., 2021) in both Introduction and Discussion. |
| Methods – Specify that the outcome variable was binary (younger vs older age). | Stated explicitly: “The binary outcome was young (40–49 years = 1) versus 50 + years = 0.” |
| Data cleaning section too detailed – consider moving range-check text to Supplementary Material. | The section was condensed; only summary statements remain in the main text, consistent with PLOS style. |
| Results – Ensure uniform table alignment and reference Table 3 explicitly when summarizing regression results. | All tables reformatted for consistent alignment and spacing. Table 3 is cited directly in the results narrative. |
| Include Cuzick trend test and per-year odds ratio. | Added: “Cuzick z = 4.56, p < 0.001; per-year logistic OR = 1.10 (95 % CI 1.06–1.14).” |
| Figure 2 caption – Summarize key findings (e.g., differences in exercise, diet, smoking). | Caption revised: “Statistically significant association (p < 0.001) observed only for exercise; no differences in diet or smoking.” |
| Discussion – Add statement on barriers among older women. | Added paragraph noting comorbidities, perceived benefit, and logistical barriers. |
| Clarify that the social-media subgroup was small (n = 94). | Added “n = 94 (1.8 %)” in both Results and Discussion with caution on precision. |
| Provide one example of culturally tailored messaging. | Added examples of mosque-based and social-media outreach strategies. |
| Highlight the practical implication of menopause-clinic integration. | Final Discussion paragraph expanded: “Embedding screening prompts in menopause clinics could leverage this touchpoint.” |
| References – Ensure all journal names are italicized and numbering consistent. | Completed; reference list fully formatted per PLOS style. |
| Formatting – Remove leftover line numbers; standardize spacing in numeric expressions (e.g., 95 % CI). | All residual line numbers removed; spacing and p-value notation standardized throughout. |

**Summary:**
All editorial comments from the 11 October 2025 decision letter have been fully incorporated into the revised manuscript. No unaddressed items remain. Reviewer #1 has confirmed satisfaction with all prior revisions.

We thank the Academic Editor for these final refinements, which have further improved the clarity and presentation of our paper.
